# Interpretation of the Yak Skin Single-Cell Transcriptome Landscape

**DOI:** 10.3390/ani13243818

**Published:** 2023-12-11

**Authors:** Qingbo Zheng, Na Ye, Pengjia Bao, Tong Wang, Chaofan Ma, Min Chu, Xiaoyun Wu, Siyuan Kong, Xian Guo, Chunnian Liang, Heping Pan, Ping Yan

**Affiliations:** 1 Key Laboratory of Animal Genetics and Breeding on Tibetan Plateau, Ministry of Agriculture and Rural Affairs, Lanzhou Institute of Husbandry and Pharmaceutical Sciences, Chinese Academy of Agricultural Sciences, Lanzhou 730050, China; zhengqingbo19@163.com (Q.Z.); yena199306@163.com (N.Y.); baopengjia@caas.cn (P.B.); wgeniust@163.com (T.W.); 13644430207@163.com (C.M.); chumin@caas.cn (M.C.); wuxiaoyun@caas.cn (X.W.); guoxian@caas.cn (X.G.); chunnian2006@163.com (C.L.); 2 Key Laboratory of Yak Breeding Engineering of Gansu Province, Lanzhou Institute of Husbandry and Pharmaceutical Sciences, Chinese Academy of Agricultural Sciences, Lanzhou 730050, China; 3 Agricultural Genomics Institute at Shenzhen, Chinese Academy of Agricultural Sciences, Shenzhen 518120, China; kongsiyuan@caas.cn; 4 Life Science and Engineering College, Northwest Minzu University, Lanzhou 730030, China; 5 Institute of Western Agriculture, The Chinese Academy of Agricultural Sciences, Changji 831100, China

**Keywords:** yak, scRNA-seq, fibroblasts, cell fate decision

## Abstract

**Simple Summary:**

Yaks can grow in extremely harsh natural environmental conditions, such as high altitude, low temperature, and hypoxia, and hair plays an important role. As an appendage of the skin, hair follicles involve the specialization of various types of cells and intercellular communication during morphogenesis. Here, we used single-cell RNA sequencing technology to identify 11 cell types from the scapular skin of yaks, constructed a transcription map of the main cells of hair follicles in the skin, and described the heterogeneity of DP cells and dermal fibroblasts in the hair follicle cycle. Our findings provide a molecular landscape of the fate determination process of dermal cell lineages. Our research provides valuable resources for further exploration of the molecular pathways involved in hair follicles.

**Abstract:**

The morphogenesis of hair follicle structure is accompanied by the differentiation of skin tissue. Mammalian coats are produced by hair follicles. The formation of hair follicles requires signal transmission between the epidermis and dermis. However, knowledge of the transcriptional regulatory mechanism is still lacking. We used single-cell RNA sequencing to obtain 26,573 single cells from the scapular skin of yaks at hair follicle telogen and anagen stages. With the help of known reference marker genes, 11 main cell types were identified. In addition, we further analyzed the DP cell and dermal fibroblast lineages, drew a single-cell map of the DP cell and dermal fibroblast lineages, and elaborated the key genes, signals, and functions involved in cell fate decision making. The results of this study provide a very valuable resource for the analysis of the heterogeneity of DP cells and dermal fibroblasts in the skin and provide a powerful theoretical reference for further exploring the diversity of hair follicle cell types and hair follicle morphogenesis.

## 1. Introduction

Hair is a highly keratinized tissue produced by mammalian hair follicles [1]. Hair follicles are a kind of skin appendage with independent functions and periodic growth and can be divided into epithelial cell groups and dermal cell groups according to cell origin and function [2,3,4]. The hair follicle is composed of a connective tissue sheath (CTS), hair bulb, inner root sheath (IRS), outer root sheath (ORS), and hair shaft (HS), and the HS is located in the center of the hair follicle and consists of three layers of cells: hair cuticle, medulla, and cortex [3,4,5]. The interaction between the epithelial cell group and dermal cell group contributes to the initial stage of hair follicle development [6]. Hair follicles undergo a continuous cycle of growth, that is, the cycle of telogen, anagen, and catagen, and depend on different cell signal exchanges between the epidermis and dermis [7,8]. Various types of cells in the hair follicle structure can control the proliferation and differentiation of hair follicle epidermal cells by secreting many signaling molecules, such as growth factors and cytokines.

Dermal fibroblasts are the most abundant cell type in the dermal layer of the skin, and their main function is to secrete the components of the extracellular matrix (ECM) to provide structural support [9]. Fibroblasts in the dermis are divided into different morphological and functional subgroups according to their spatial localization [9,10,11]. Fibroblasts connected to the epidermal basement membrane in the dermis are called papillary fibroblasts (PFs), and those located in the lower reticular dermis fibroblasts are called reticular fibroblasts (RFs). Driskell et al. [11] used lineage tracing to trace the origin of fibroblasts at different levels of the dermis in a mouse embryonic model and found that dermal fibroblast progenitor cells differentiated into the PF lineage and RF lineage at 16.5 weeks in mouse embryos. The process of skin aging mainly affects the dermis, the spinous process structure at the junction of the epidermis and dermis is significantly reduced, and the dermis shrinks [12,13,14]. The lineage relationship between all fibroblast subtypes in the dermis plays an important role in development, homeostasis, aging, wound repair processes, and maintaining the healthy state of the dermal microenvironment [11,15,16]. In recent years, single-cell RNA sequencing (scRNA-seq) has provided unprecedented insights into the specific transcription profiles of cell subsets in the skin, particularly regarding fibroblasts and their relationship with other cells in the dermis. Guerrero-Juarez et al. [17] used scRNA-seq technology to explore the diversity of skin wound fibroblasts and obtained 12 fibroblast clusters, some of which may be in a continuous differentiation state towards the contractile phenotype; some cell clusters may represent different fibroblast lines, and some subsets of cell lines express hematopoietic marker genes. Vorstandlechner et al. [18] used scRNA-seq to analyze the heterogeneity of human skin fibroblasts and identified six FB subgroups and found that the newly identified FB subgroups did not overlap with the markers commonly used to identify papillary and reticular fibroblasts. Thompson et al. [19] studied the accessibility characteristics of myofibroblast markers in neonatal fibroblasts and showed that different fibroblast subtypes, such as PF and RF, can support myofibroblast status.

Dermal papilla (DP) cells control the formation and size of hair follicles and are the control center of hair follicle growth and the hair follicle cycle. The DP is considered the central player in hair follicular cycle regulation, being triggered by paracrine signaling, and it serves as a reservoir for multipotent stem cells and various growth factors, participating in the regulation of hair follicle development and growth [20]. The number of DP cells in hair follicles and the size of the dermal papilla area formed by them are key to distinguishing different types of hair follicles [21]. The number of DP cells in different hair follicle cycles affects the occurrence of hair follicle morphology [22]. During the late anagen phase, adjacent cells to DP cells undergo apoptosis, causing the hair follicle cycle to enter catagen; the hair follicle enters telogen, while DP cells remain in dense spherical form; as the follicle cycle transitions back to anagen, DP cells become loose, engulfed by new hair bulbs and become slender [5]. Rompolas et al. [23] removed DP cells from mouse skin hair follicles during telogen via two-photon confocal microscopy and found that hair follicles that removed DP cells could not enter anagen, while hair follicles with intact DP cells could enter anagen normally. When the number of DP cells decreased, the process of entering anagen in hair follicles was delayed.

The yak is mainly distributed in the Qinghai-Tibet Plateau and its surrounding areas. It is a valuable livestock and poultry variety resource in the Qinghai-Tibet Plateau [24]. Yaks have thick skin and dense hair, which is one of the important reasons for their ability to adapt to cold and strong-ultraviolet environments. The yak has a unique mixed hair structure; its hair is composed of coarse hair, two-type hair, and fluff, and yak fluff is also a precious textile material [25]. The hair follicles of the yak show obvious seasonal periodic changes, accompanied by the growth and shedding of villi. Hair follicle morphogenesis is the result of the interaction of many cell types. Compared with other common villus-producing animals, there are few studies on different cells during the hair follicle cycle development of the yak. The study of the heterogeneity of dermal cells in the telogen and anagen phases of hair follicles will help to reveal the mechanism of dermal cell interaction and signal communication, which is significant for understanding the underlying processes in hair follicle biology.

To systematically understand the diversity of skin dermal cells, and to fully understand the pedigree heterogeneity of DP cell and dermal fibroblasts in the dermis and the fate regulatory mechanism of different cell subtypes, here, we used scRNA-seq technology to identify 18 cell clusters and 11 cell types from the scapular skin of the yak, constructed the main cell transcription map in hair follicles, and focused on the regulatory mechanism of fate determination in the specialization process of different fibroblast subsets. This work not only enriches people’s understanding of the morphogenesis of hair follicles in different cycles of yak, but also clarifies the differences in fibroblast subsets and their mechanism of action in the maintenance of skin homeostasis and provides a theoretical reference for further exploring the molecular mechanism of hair follicle cycle development in the yak.

## 2. Materials and Methods

### 2.1. Experimental Animals

The yak skin samples used in this study were all derived from the Tianzhu White Yak Breeding Farm in Wuwei City, Gansu Province. We selected three representative female yaks with a body weight of 2–3 years old consistent with the average body weight of their breeds and good health. According to the characteristics of yak hair follicle cycle development, we collected skin tissue of the yak scapula for the preparation of single-cell suspension during hair follicle anagen and telogen [1]. Before collection, the hair on the shoulder blade of the yak was trimmed by using elbow scissors, 2% lidocaine was injected subcutaneously for local anesthesia, and then the skin tissue was transferred using a skin sampler. From the collected skin, we removed as much subcutaneous fat tissue as possible, disinfected the sample with 75% alcohol, and washed with D-PBS to remove the alcohol. After treatment, the tissue was immediately stored in tissue protection solution and brought back to the laboratory for suspension preparation, and the other part was placed in paraformaldehyde for verification experiments.

### 2.2. Single-Cell RNA Sequencing of Yak Hair Follicles

The preparation of single-cell suspension and scRNA-seq analysis were performed according to the previous report [26]. After mixing the cell suspension of three samples into a mixture, it can be used for sequencing when the cell survival rate is ≥85%. The formation of single-cell GEM was performed using 10 × Genomics’ Chromium Single Cell 3’v3 Gel Beads kit. According to the instructions of the Chromium Single Cell 3’v3 Gel Beads kit, PE150 sequencing was performed using the Illumina NovaSeq 6000 platform. STAR in CellRanger was used to compare the readings with the splicing recognition of the reference genome. After CellRanger was completed, the next step is to cluster the scRNA-seq data. In this study, the “filtered_gene_bc_matrices” file generated via CellRanger analysis was read using Seurat in R environment (R version: 4.0.5), and then low-quality cells were filtered using the FilterCells function for different samples. The NormalizeData function was used to standardize the data, and then the expression matrix was subjected to dimensionality reduction clustering and cell type identification.

### 2.3. Construction of the Cell Differentiation Trajectory

To further analyze specific cell clusters and construct the differentiation trajectory of DP cells and fibroblast lines, we first used Seurat’s “SubsetData” function to extract fibroblast cell lines and DP cell clusters. Monocle (v2.18.0) was used to sort individual cells along pseudotime according to the official tutorial. To perform quasitime sorting on specific cell types, we use the “newCellDataSet” function in Monocle to construct Monocle objects. The state was set according to the cell cluster label identified using seurat, and the branch-specific expression genes were prepared using the “BEAM” function. The heat map was drawn using Monocle’s “plot_genes_branched_heatmap” function, and the genes with qval < 10^−4^ were regarded as input genes, after which the gene clusters were further divided into four clusters according to the k-means.

### 2.4. GO Enrichment Analysis

With the help of Metascape (http://metascape.org/gp/index.html#/main/step1) (accessed on 26 June 2023) online software, GO analysis of characteristic genes was performed. The gene names were transformed into gene IDs through the R package org.HS.eg.db (v3.12.0) annotation package, and the possible pathways were enriched with the clusterProfiler (v3.18.1), ggplot2 (v3.3.5), and enrichplot (v1.10.2) packages.

### 2.5. Cell Communication Analysis

CellChat (v1.5.0) was used to analyze intercellular communication. CellChat calculates the communication probability at the signaling pathway level by summarizing the communication probability of all ligand–receptor interactions associated with each signaling pathway. A circle plot was used to show the number and strength of interactions between cells. A bubble diagram was used to display the interaction ligand–receptor pairs, and Seurat was used to plot the gene expression distribution of signal genes related to the signaling pathway.

### 2.6. Immunofluorescence Analysis

The skin tissue was taken out from the 4% paraformaldehyde fixative. The skin tissue was trimmed with a scalpel, and the dehydration box was placed in a dehydrator (Diapath, Martinengo, BG, Italy) for dehydration and waxing leaching with different concentrations of gradient alcohol (Sinopharm Chemical ReagentCo., Ltd., Shanghai, China) and xylene (Sinopharm Chemical ReagentCo., Ltd., Shanghai, China). Then the melted wax was placed in the embedding box, and the tissue was taken out from the dehydration box and placed in the embedding box before the wax solidified and cooled at −20 °C. The tissue block was cut into 4 μm slices using a slicing machine (Shanghai Leica Instrument Co., Ltd., Shanghai, China), and then the slices were unfolded in 40 °C warm water. The tissue was fished up with a glass slide and baked at 60 °C. The sections were dewaxed in xylene, alcohol, and distilled water to water, and EDTA antigen retrieval buffer (Servicebio, Wuhan, China) was used for antigen repair. After natural cooling, the sections were placed in PBS (Servicebio, Wuhan, China) for washing. During the immunofluorescence single-labeling staining experiment, the slides were incubated overnight with the primary antibody at 4 °C, then placed in PBS and washed, and the corresponding second antibody was added and incubated in darkness at room temperature for 50 min. In immunofluorescence homologous double-labeling staining experiments, the first primary antibody needs to be added. After incubation at 4 °C overnight, the HRP-labeled secondary antibody of the corresponding species was added to cover the tissue and incubate at room temperature for 50 min. After washing the glass slides, TSA was added and incubated in the dark for 10 min, and then heated after washing. After that, the second primary antibody was added and incubated overnight at 4 °C. After washing, the fluorescent secondary antibody of the corresponding species of the primary antibody was added and incubated for 50 min. After incubation with primary and secondary antibodies, the slides were nuclear-stained with DAPI (Servicebio, Wuhan, China), autofluorescence quencher (Servicebio, Wuhan, China) was added for 5 min, and the sections were rinsed with running water for 10 min. Finally, the slides were examined using a fluorescence microscope (Nikon, Tokyo, Japan). The primary antibodies MSX1, VIM, and DLX3 were from Bioss (Bioss, Beijing, China).

## 3. Results

### 3.1. Single-Cell Data Set Quality Statistics

Using scRNA-seq, 26,573 single cells were obtained from the scapular skin of yaks. The data quality of each sample is shown in Table 1. The valid barcode of two samples is higher than 95%, and the genome reads mapped is greater than 85%. The number of cells detected in the telogen sample was 14,573, and the number of genes detected was 17,796; the number of cells detected in the anagen sample was 12,000, and the number of genes detected was 18,420. The overall data quality is at an ideal level.

### 3.2. Identification of Main Cell Types and Analysis of Characteristic Gene Expression

**Figure 1:**
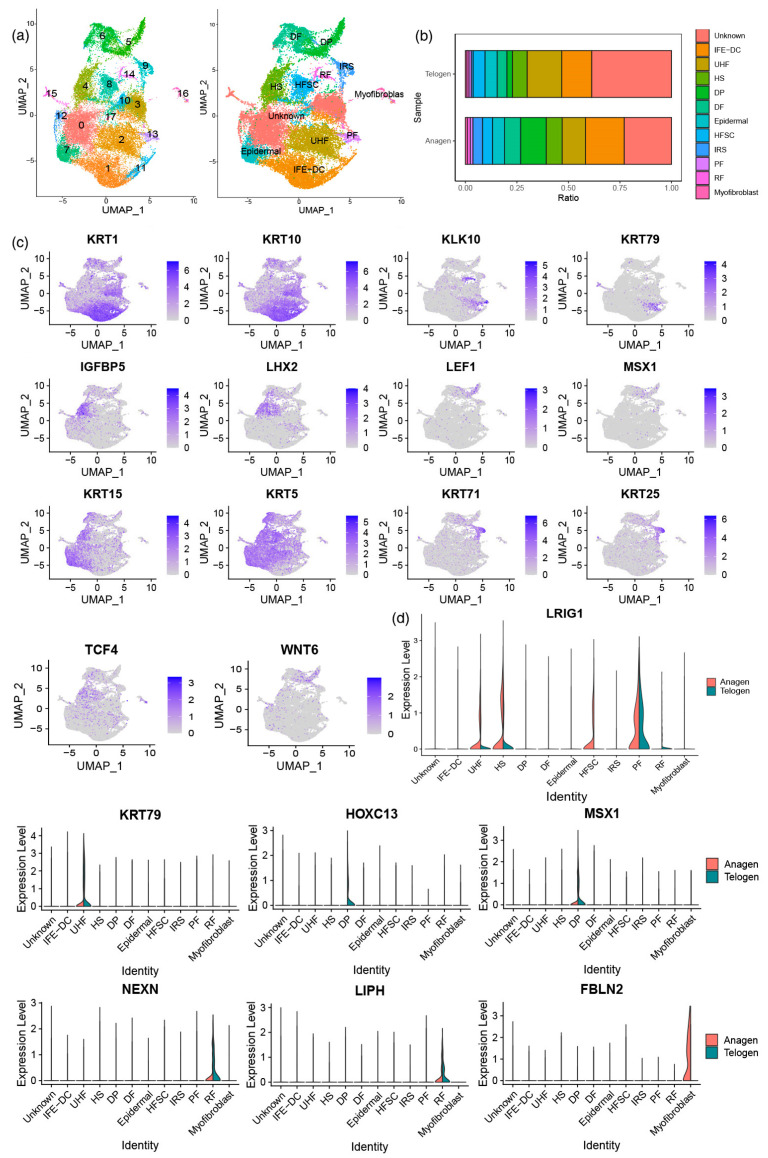
Single-cell UMAP cluster analysis. (**a**) Data integration analysis and identification of major cell types; (**b**) main cell type proportions during telogen and anagen; (**c**) the expression of different types of cell-specific marker molecules in all cells; (**d**) the expression of characteristic genes in different cell types.

To construct the transcription map of yak skin tissue, we integrated the hair follicle telogen and anagen data, created a gene cell expression matrix, and reduced the dimension via UMAP clustering to determine 18 clusters (Figure 1a). We obtained 18 clusters of cell types. According to the comparison of the marker genes in the published papers with the specific genes expressed in each cluster, 11 cell types were identified. It was found that IFE-DC and DP cells were highly expressed during anagen (Figure 1b). Furthermore, cluster 1 and 11 (interfollicular epithelium differentiation cell, IFE-DC) marker genes KRT1 [27], KRT10 [27], and SBSN [27] were also found. Cluster 2 expressed the UHF cell marker genes KLK10 [28] and KRT79 [28]. Cluster 4 expressed the HS cell marker genes IGFBP5 [29] and LHX2 [30]. Cluster 5 expressed the DP cell marker genes LEF1 [31], MSX1 [32], and HOXC13 [32]. Cluster 7 expressed the epidermal cell lineage marker genes KRT5 [27] and KRT15 [33,34]. Cluster 8 expressed hair follicle stem cell (HFSC) cell marker genes SOX9 [35,36,37,38] and TCF4 [38]. Cluster 9 expressed the IRS cell marker genes KRT71 [39] and KRT25 [40]. Figure 1c shows the expression of some marker genes. Cluster 6 expressed dividing the fibroblast (DF) cell marker genes WNT6 [19] and NFIC [19]. Cluster 13 expressed the PF cell marker genes LRIG1 [11] and SCEL [19]. Cluster 14 expressed the RF cell marker genes LIPH [19], MGST1 [19], NEXN [13], and TPM1 [13]. Cluster 16 expressed the myofibroblast marker genes TGFBR2 [19] and FBLN2 [17]. Figure 1d shows the expression of some marker genes in different cells during anagen and telogen. The expression of FBLN2 in myofibroblasts was mainly in hair follicle anagen.

**Figure 2:**
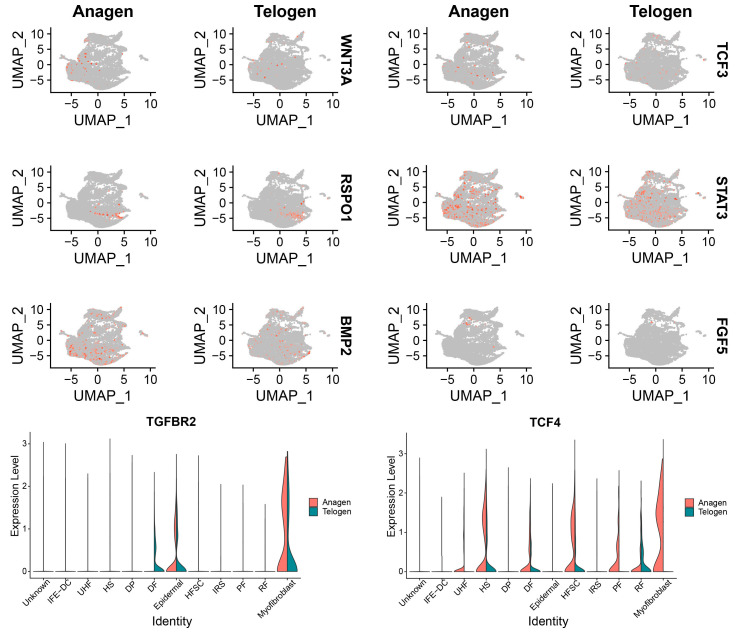
The expression of key genes in anagen and telogen of hair follicles.

After identifying the cell types of yak hair follicles, we observed expression changes in some characteristic genes in hair follicles during telogen and anagen, and found that genes such as STAT3 and TCF4 were upregulated during anagen (Figure 2). Current studies have found that SHH, WNT/β-catenin/LEF1, and STAT3 signals are activated during the transition of hair follicles from telogen to anagen [3,41,42], indicating that STAT3 and other genes play a very important role in the transition of the hair follicle cycle in early anagen. The expression level of RSPO1 was significantly upregulated during the transition from telogen to anagen in mouse dorsal hair follicles [43]. We found that RSPO1 was expressed in yak hair follicles during hair follicle anagen and telogen. In a study of humans, it was found that RSPO1 was mainly expressed in dermal fibroblasts, and the expression of RSPO1 was not detected in cultured keratinocytes [44,45]. The expression level of TGFBR2 in myofibroblast cells was increased during the transition from telogen to anagen. Furthermore, we found that WNT3A was also up-regulated during hair follicle anagen. Guo et al. used qRT-PCR and Western blotting to detect the telogen and anagen of mouse hair follicles and found that WNT3A had the highest expression in the anagen of hair follicles and that the expression in telogen was weak [46].

### 3.3. Fate Specialization Process of Dermal Fibroblasts

**Figure 3:**
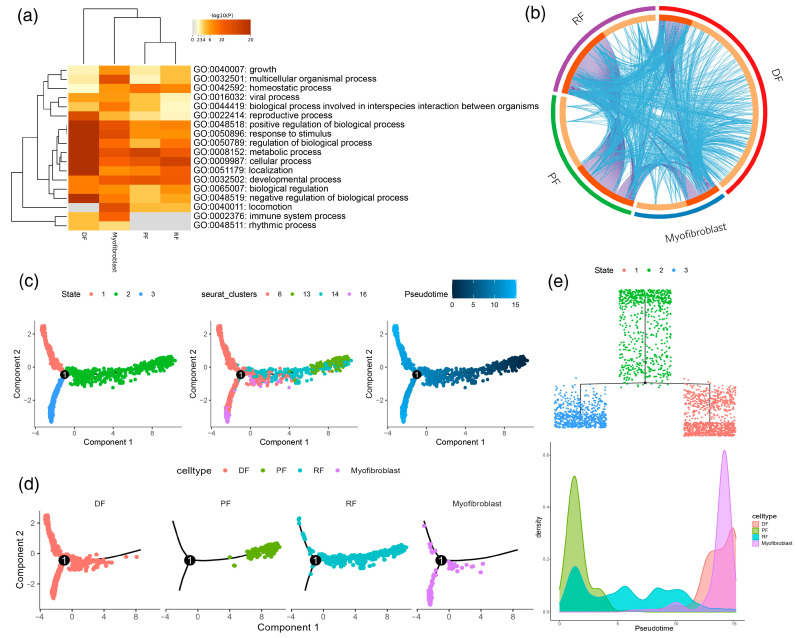
Comparison and pseudotiming analysis of GO pathways enriched in different clusters of fibroblast lineage. (**a**) Comparison of GO pathways enriched in different clusters of fibroblast lineage; (**b**) The Circos map shows the GO terms shared by the four gene sets; (**c**) construction of the pseudotime differentiation trajectory of fibroblasts; (**d**) cell branch trajectory map; (**e**) fibroblast tree diagram and cell density diagram.

Through UMAP dimensionality reduction cluster analysis, the gene expression profiles of different types of cells in yak hair follicle cycle changes were described in detail, and a series of cell type-specific genes were obtained. The results show that four different clusters were obtained via UMAP clustering for fibroblasts, which were clusters 6, 13, 14, and 16. Further GO enrichment analysis showed that the enriched GO pathways of clusters 13 and 14 were very similar, including response to stimulus, developmental process, and locomotion, but immune system process and rhythmic process were not enriched. For cluster 6, the characteristic genes expressed in these cells were significantly enriched in positive regulation of biological process, response to stimulus, regulation of biological process, metabolic process, cellular process, localization, and negative regulation of biological process, which shows the response of fibroblasts to external stimuli and the regulation of cell cycle, indicating a high degree of differentiation and tissue specificity (Figure 3a). At the same time, the results of the Circos map also show that there are many of the same genes and coenriched pathways between these different clusters, which also suggests that they are similar (Figure 3b). Based on the continuity of cells in the clustering results, we constructed pseudotime trajectories for four fibroblast-related cell populations to analyze the dynamic expression of genes during the differentiation of specific cell types. Using quasitime ordination analysis, one branch and three different states were obtained according to the gene expression pattern (Figure 3c), in which PF and RF were mainly concentrated in state 2, and myofibroblasts were mainly concentrated in state 3 (Figure 3d). The results show that the density of PF was high in the early stage and then gradually decreased, while the density of DF and myofibroblast was higher in the later stage (Figure 3e).

**Figure 4:**
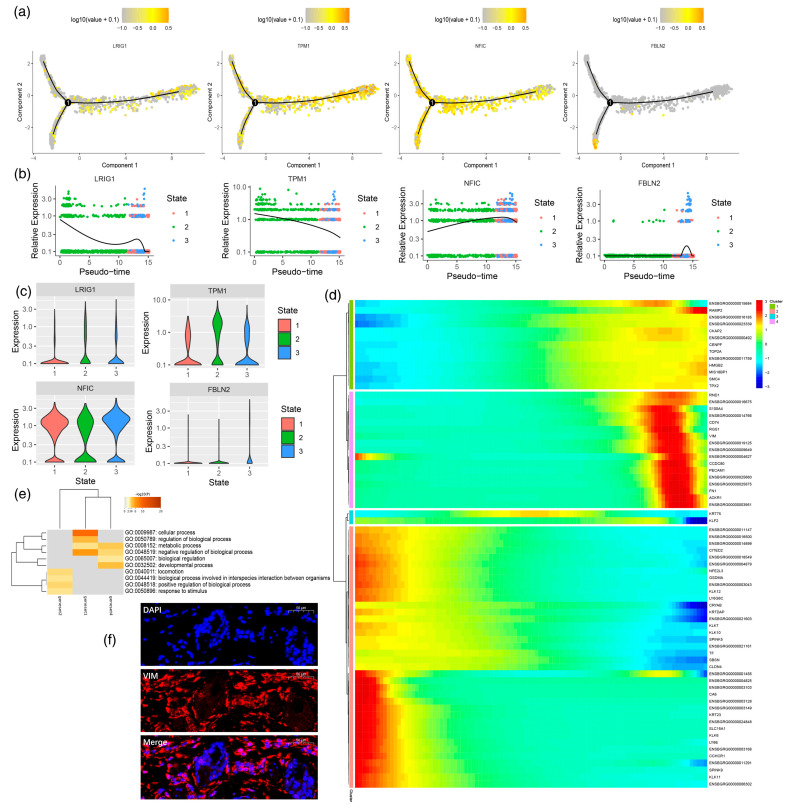
Molecular characteristics in the process of fibroblast specialization. (**a**) Differential expression of characteristic genes in branches; (**b**) changes in the expression of characteristic genes over time; (**c**) the expression of characteristic genes in the lower state; (**d**) gene dynamic changes in the process of cell specialization; (**e**) comparison of GO terms in different gene sets; (**f**) immunofluorescence analysis.

In addition, based on the quasitime sorting analysis, we analyzed the expression of four marker genes in the differentiation trajectory of fibroblasts, and found that the expression levels of TPM1 and NFIC were higher than those of FBLN2 during the differentiation process (Figure 4a). To further compare the changes in gene expression in different cell branches in different states, a series of genes were selected for differential analysis. For genes such as LRIG1 and TPM1, which are highly expressed before branching, the expression level is high in state 2 and low in state 1. The expression level of NFIC was upregulated in the process of cell specialization. FBLN2 was mainly concentrated in state 3, and its expression level was significantly lower than that of LRIG1, TPM1, and NFIC. We constructed a dynamic differentiation heatmap of differential genes (Figure 4b–d) and obtained four different gene sets according to their expression patterns. At the beginning of differentiation, it was mainly the specialization process of PF and RF cells. According to the pseudotemporal differentiation heatmap, CA6, KRT23, SLC15A1, KLK6, LY6E, CCHCR1, and GSDMA were highly expressed at this stage. For VIM, RGS1, CD74, and S100A4, the expression level was first upregulated and then downregulated. According to the gene expression matrix in the cluster heatmap results, the genes were divided into different genomes according to the expression pattern. Gene sets 1, 2, and 4 were mainly enriched in the cellular process, regulation of biological process, metabolic process, negative regulation of biological process, developmental regulation, locomotion, biological process involved in interspecies interaction between organisms, positive regulation of biological process, and response to stimulus (Figure 4e). In this study, the skin tissue of yaks was stained and analyzed via immunofluorescence (Figure 4f). The results show that VIM-positive cells were mainly concentrated in fibroblasts around hair follicles.

### 3.4. The Fate Specialization Process of Dermal Papilla Cells

**Figure 5:**
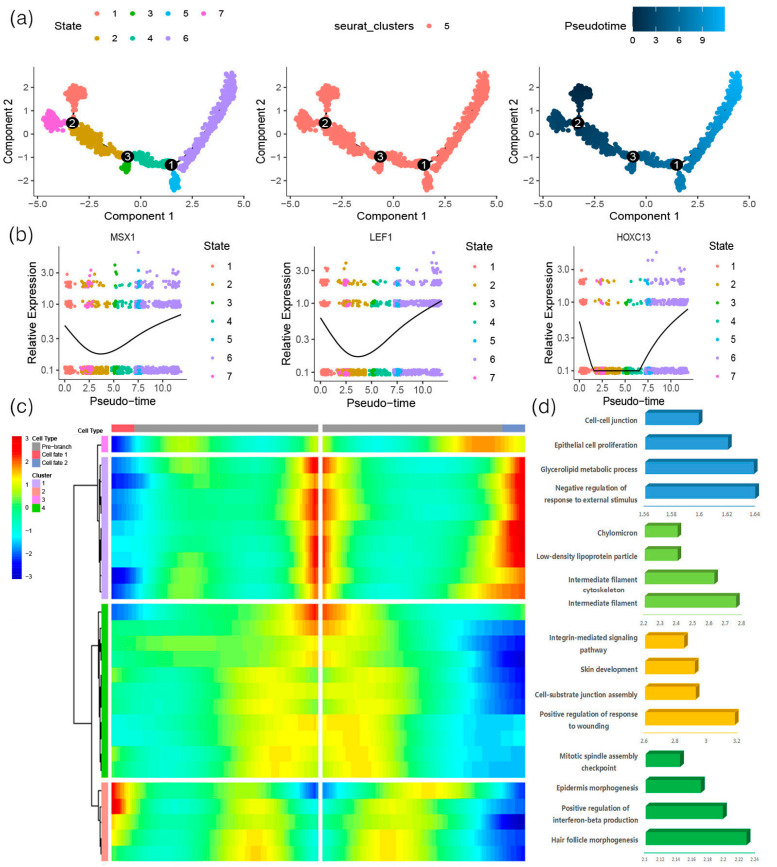
DP cell differentiation trajectory and dynamic changes in the branch 1 gene. (**a**) Construction of the DP pseudotime differentiation trajectory; (**b**) changes in the expression of LEF1, MSX1, and HOXC13 in pseudotime; (**c**) heatmap displaying the branch 1 expression pattern; (**d**) GO enrichment analysis of differentially expressed genes.

DP cells were extracted from Seurat, and the Monocle algorithm was used to analyze the pseudotime differentiation trajectory of DP cells. We divided the specialization process into three branches. We analyzed the expression changes of DP marker genes in each state (Figure 5a). The expression levels of LEF1, MSX1, and HOXC13 in DP cells were first downregulated, then upregulated, and were highly expressed in state 6, among which HOXC13 was low in state 2, 3, and 4 (Figure 5b,c). Subsequently, we divided the branch-1-specific differential genes into four gene sets and performed expression analysis to further analyze the mechanism of DP cells in the hair follicle cycle transition process and the functional enrichment in the intermediate cell specialization process. The results show that gene set 1 was enriched in the negative regulation of external stimulus response, glycerolipid metabolism, epithelial cell proliferation, and cell–cell junction. Gene set 2 was enriched in intermediate filaments, intermediate filament cytoskeleton, low density lipoprotein particles, and chylomicrons. Gene set 3 was enriched in positive regulation of nociceptive response, cell–substrate junction assembly, skin development and integrin-mediated signaling pathway. Gene set 4 was enriched in hair follicle morphogenesis, positive regulation of interferon-β production, epidermal morphogenesis, and mitotic spindle assembly (Figure 5d).

**Figure 6:**
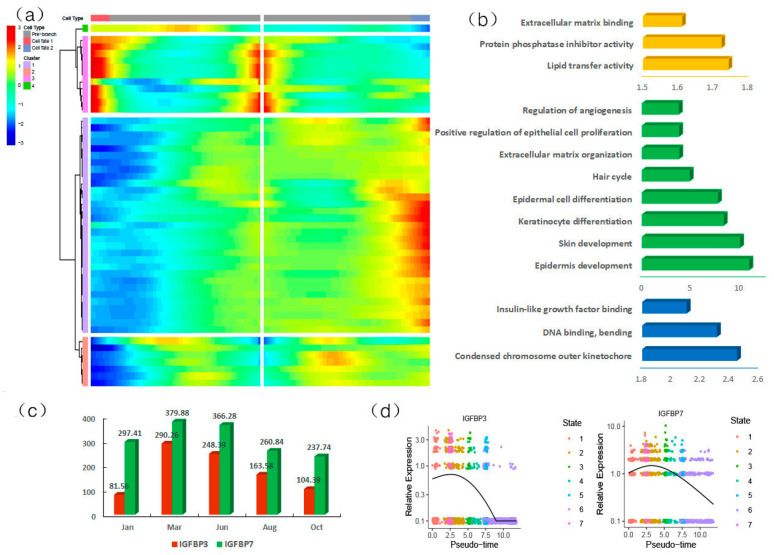
Analysis of DP cell second branch cell fate. (**a**) Heatmap displaying the branch 2 expression pattern; (**b**) GO enrichment analysis of differentially expressed genes; (**c**) expression of the IGFBP3 and IGFBP7 genes in different stages of yak hair follicle cycle development; (**d**) changes in the expression of IGFBP3 and IGFBP7 in pseudotime.

We analyzed the branch-2-specific differential genes (Figure 6a). The results of enrichment analysis show that the second gene set was mainly enriched in lipid transfer activity, protein phosphatase inhibitor activity, and extracellular matrix binding. GO enrichment of the third gene set shows that it was mainly concentrated in pathways related to hair follicle development, such as epidermal development, extracellular matrix tissue, skin development, keratinocyte differentiation, hair cycle, epidermal cell differentiation, positive regulation of epithelial cell proliferation, and regulation of angiogenesis (Figure 6b). Among them, genes such as KRT17, SPINK5, and SOX9 are related to the cycle of the hair follicle cycle, and genes such as KRT17 and SOX9 are related to cell proliferation and hair follicle cycle development in the growth period. The high expression of these genes is more conducive to the regeneration of anagen hair follicles and the maintenance of cell homeostasis in degenerative hair follicles [47]. The fourth gene set was mainly enriched in the extrachromosomal kinetochores, DNA binding bending, and insulin-like growth factor binding, while IGFBP7 was involved in the related pathways of insulin-like growth factor binding. As a polypeptide growth factor, insulin-like growth factor plays an important role in cell anti-apoptosis, cell mitosis, and hair follicle dermal cell proliferation and differentiation [48]. Lee et al. [49] found that when follicular-keratinocyte-conditioned media (FKCM) was supplemented as a medium, proteins such as IGFBP7 had the function of enhancing hair induction, which enhanced the hair production of rat vibrissa DP cells and activated β-catenin and BMP signaling pathways. IGFBP3 is expressed in the dermal papilla, and its mRNA expression level is significantly increased in the early stages of degeneration and telogen compared to anagen [50]. We obtained data on different periods of the yak hair follicle cycle development from NCBI: PRJNA550233. The expression of IGFBP3 and IGFBP7 in different periods of the yak hair follicle cycle was observed via joint analysis. The results show that the fragments per kilobase of exon model per million fragments mapped (FPKM) values of IGFBP3 and IGFBP7 in the catagen and telogen groups were higher than those in the anagen group (Figure 6c). By further analyzing the expression of IGFBP3 and IGFBP7 in different states, it was found that the expression levels of IGFBP3 and IGFBP7 in dermal papilla cells were upregulated first and then downregulated, and the expression level was the lowest in state 6 (Figure 6d), which further indicates that the expression level of IGFBP3 and other genes in telogen were higher than those in anagen, which is consistent with the reported conclusions.

**Figure 7:**
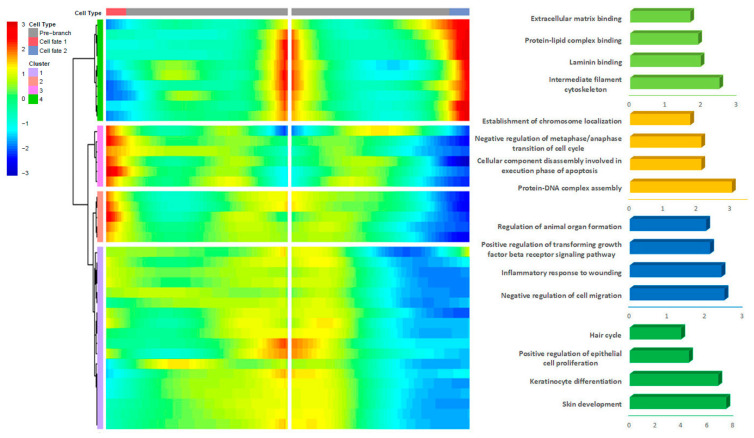
Analysis of the cell fate of the third branch of DP cells.

We analyzed the expression of branch-3-specific differential genes (Figure 7). The results show that the first gene set was mainly enriched in intermediate filament cytoskeleton, laminin binding, protein–lipid complex binding and extracellular matrix binding. The second gene set was mainly enriched in protein–DNA complex assembly, cell component decomposition involved in the execution phase of apoptosis, establishment of chromosome localization, and negative regulation of mid/late transition of cell cycle. Some researchers have analyzed the expression patterns of hair follicle cycle genes to show the differences in DP cells, and 825 differentially expressed genes were obtained from DP cells in the anagen phase and the telogen phase. Differential genes have functions such as cell cycle control, chromosome distribution, and cell division. It was found that secondary hair follicles in the middle stage of hair growth can not only induce the deformation of villi and the stagnation of villi growth, but also resist the apoptosis of hair follicles themselves and prepare for hair growth [51,52]. The third gene set was mainly enriched in the negative regulation of cell migration, the inflammatory response to wounds, the positive regulation of transforming growth factor β receptor signaling pathway, and the formation of animal organs. The fourth gene set was mainly enriched in items such as skin development, positive regulation of epithelial cell proliferation, keratinocyte differentiation, and hair cycle. The GO enrichment results of the fourth gene set were similar to those of the third gene set of the second branch.

### 3.5. CellChat Analysis

**Figure 8:**
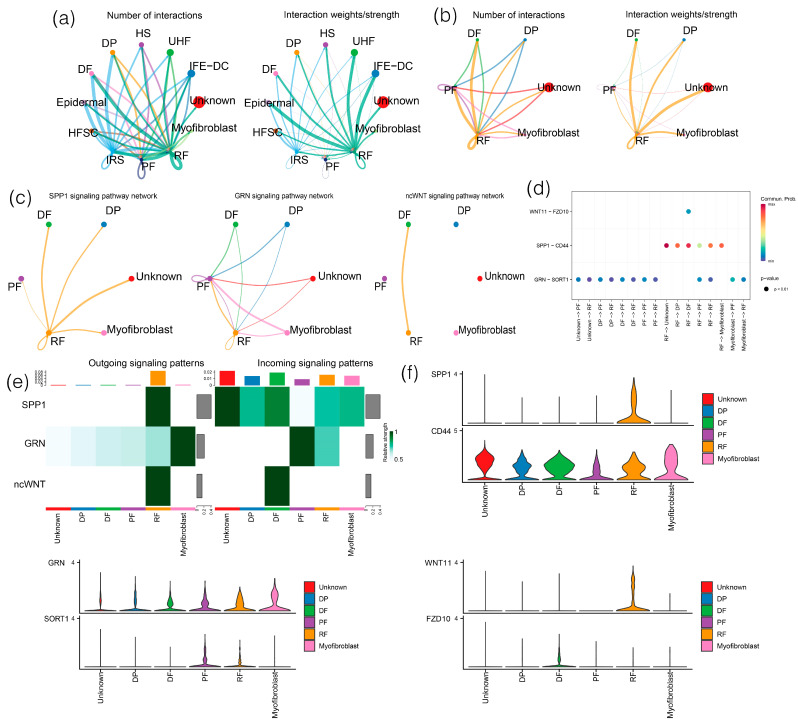
Reasoning of intercellular communication in hair follicles. (**a**) Figures of the number and weight of cell–cell communication interactions of identified cell types: (**b**) intercellular communication between fibroblast types; (**c**) signaling pathway network diagram; (**d**) signaling-pathway-mediated intercellular communication; (**e**) output and input signal recognition of different cell groups. (**f**) distribution map of signal gene expression.

To understand cell interactions in the dermal fibroblast lineage, Cellchat [52] was used to infer the number and strength of interactions between different cell populations during the telogen and anagen of yak hair follicles. The results show that most of the interactions occurred between fibroblasts, IFE-DC, and IRS (Figure 8a). Then, the cell interaction between different cell types of fibroblasts was explored by the expression of ligand–receptor pairs. A cell communication analysis showed that RF has a high number and intensity of interactions with other cell types. Among them, the number of cell interactions between RF and PF and DF was greater, but the interaction intensity between RF and DF, DP, and myofibroblasts was significantly higher than that between RF and PF (Figure 8b). CellChat found multiple signaling pathways in the ligand–receptor pair of the fibroblast lineage, including the ncWNT, SPP1, and GRN pathways. In addition, the ncWNT signaling pathway from RF cells to DF cells was enriched, and the GRN signaling pathway was most abundant from myofibroblasts to PF cells (Figure 8c). Further analysis of the interaction of ligand–receptor pairs found that other cell types act on RF cells mainly through GRN-SORT1 ligand–receptor pairs. RF cells interact with other cell types mainly through SPP1-CD44 and GRN-SORT1 for cell communication (Figure 8d). Previous CellChat analysis of the ncWNT signaling network showed that ligands (WNT5A) and a fibroblast population mainly drive fibroblast-to-fibroblast, fibroblast-to-endothelial, and a small amount of fibroblast-to-medullary signals [53]. To reveal the effect of signaling pathways on intercellular communication, the interaction intensity of the three signaling pathways was demonstrated. Compared with other cells, RF cells were the most important cells for SPP1 signaling pathway output. In addition, the study also observed that the expression of the SPP1 and WNT11 genes was relatively high in the RF subgroup, and noncanonical WNT signals such as WNT11 were highly expressed in RF, suggesting that RF cells may communicate with DF cells through noncanonical WNT signaling pathways (Figure 8e,f).

**Figure 9:**
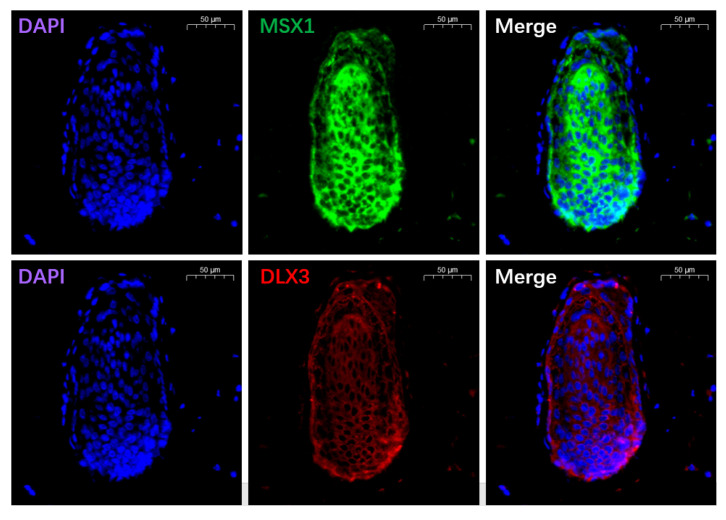
Immunofluorescence analysis of key marker proteins in yak hair follicles.

In this study, immunofluorescence was used to analyze yak skin (Figure 9). The results show that MSX1 positive cells were expressed in the tissues of the hair bulb, especially in the lower DP cells. The expression level of DLX3 positive cells in DP cells was significantly lower than that of MSX1. Furthermore, we found that DLX3 also had a certain level of expression in the root sheath.

## 4. Discussion

Hair follicles are micro-organs with high cell heterogeneity. Hair follicle development starts from the embryonic period and eventually develops into an epithelial cell lineage and a dermal cell lineage composed of a variety of cells, and the whole development process is regulated by a variety of regulatory factors. Understanding the regulatory mechanism of hair follicle development and periodic growth of hair follicles is of great significance for the breeding of villus animal varieties and the improvement of villus yield. Fibroblasts are the most abundant cell type in the dermis of the skin. Thompson et al. [19] obtained 10 and 8 fibroblast clusters from scRNA-seq and scATAC-seq datasets, respectively, revealing that reticular fibroblasts are expected to follow the adipogenesis process, while neonatal fibroblasts may differentiate into the dermis. At the same time, during embryonic development, fibroblasts proliferate to fill the dermal structure and form dermal condensates along the regeneration trajectory [54,55,56]. Salzer et al. [57] found that due to development and aging, the skin maturation process may reduce the plasticity of the fibroblast population, and they found that upper dermal fibroblasts may easily establish cell contact and conduct signal transduction with adjacent cells, while lower dermal fibroblasts express more transcripts related to the extracellular matrix. Kim et al. [58] revealed the postnatal maturation trajectory of PFs via single-cell transcriptomics, and obtained cell clusters of the fibroblast lineage, including PF, RF, DP, and the dermal sheath, and two different differentiation trajectories in the upper fibroblast lineage were defined: Fb1 → Fb2 → Fb3 (FB maturation pathway) and Fb1 → Fb2 → Dp1 → Dp2 (DP regeneration pathway). There is a certain correlation between fibroblasts and hair follicles located in the epithelium and dermis in skin with rich hair [59]. PF has the special signal characteristics needed for hair follicle morphogenesis and coordination of hair growth [60]. At present, the internal mechanism of the spontaneous loss of regenerative capacity of dermal fibroblasts is still largely unknown. The differentiation of fibroblasts during the hair follicle cycle still needs further study.

At the beginning of telogen, there was no obvious expansion, apoptosis, or differentiation of hair follicles, and DP cells migrated to the bulge area near the hair follicle stem cells [61,62]. In telogen, the hair shaft is mature, and the distance between the DP and the hair bulb is shortened to the maximum extent. The transition from telogen to anagen is considered to be the activation of stem cells in hair follicle enlargement, the activation of dermal papilla cells, the interaction between the epidermis and the dermis, and the construction of the hair bulb, and the regeneration of secondary hair buds is the beginning of a new round of hair follicle cycle [63,64]. DP cells stop growing in the bulge area, so that DP cells and stem cells can interact directly. DP cells are essential for the regulation of stem cells and the initiation of the hair cycle. Studies have found that when the stem cell activator reaches a specific threshold, the growth period is initiated [5].

DP cells are the key cell type of hair follicle cycle transition, as they can coordinate mesenchymal–epithelial interactions and regulate hair follicle development and cycle transition [65]. Therefore, HFSCs and DP cells are necessary to initiate the first step of the new hair cycle [5,66]. The DP acts as the physical niche of progenitor cells during anagen and telogen, provides an opportunity to guide the proliferation of progenitor cells and the differentiation of their derivatives by inducing signal transduction [67]. Sun et al. [68] proposed that the DP sends signals to stem cells in the bulge region to initiate a new anagen. The DP expresses Wnts, R-spondins, FGFs, and Noggin, which can promote hair follicle growth and help initiate hair follicle regeneration [69,70]. DP cells can induce the transition from hair follicle anagen to catagen phase, and hair follicles in DP cells lacking β-catenin will enter the catagen in advance [71]. At present, the cycle transition at different stages of hair follicle development requires fine regulation between cell types such as DP. Therefore, if the molecular characteristics of DP cells during the transition of yak hair follicle cycle can be revealed in detail, it is of great significance to better understand the regulatory mechanism of the hair follicle cycle. In this study, scRNA-seq technology was used to analyze the molecular characteristics of DP cells in yak hair follicles from telogen to anagen. The expression profiles of three branch points were obtained, and the functional description of each lineage was described in detail. These findings provide new insights into hair regeneration.

During the transition of hair follicles from telogen to anagen, DP cells can secrete growth factors to participate in the regulation of hair follicle stem cell status and promote the cycle of hair follicles [72]. Chi et al. [73] proposed that the number of DP cells is directly related to the ability of hair follicles to initiate new hair growth: when the number of DP cells decreases below a specific threshold, hair follicles cannot initiate new hair cycles, but when a sufficient number of DPs is retained, hair follicles still have the ability to re-enter the growth phase. In mice, 20% of the hair follicles produce serrated hair in the primary integument, and more secondary hair types are produced in the second growth period, while more DP cells are needed for complex hair types, indicating that there may be functions related to hair type conversion in DP [21]. Hair follicle catagen is a dynamic transition period of the hair follicle cycle from anagen to telogen, which is regulated by molecules including FGF5 growth factor [74] and TGF-β pathway members [75,76]. IGF1 [77], PDGFB [78], WNT3A, WNT7A [79], and TAK1 [80] can maintain the anagen of hair follicles, of which only IGF1 [77] and PDGFA [81] are expressed in dermal papilla cells. There are two important signal transductions from the telogen to the anagen hair follicle cycle, in which β-catenin is the core pathway, and the switch that restarts the anagen hair follicle is the transient activation of β-catenin signaling [82,83,84,85]. It has also been found that RSPO1, similar to β-catenin, can also activate hair follicles into the anagen, but its mechanism is related to the activation of hair follicle stem cells [43]. BMP signaling plays an important role in the development of DP and the regulation of signal transmission [86]. In addition, it plays a role in inhibiting stem cells in the carina area [63]. BMP signaling can inhibit the transformation of hair follicles from telogen to anagen [87]. We found that the content of BMP2 was higher in the telogen of yak hair follicles. It is worth discussing that whether there are similarities and differences in the mechanism of action of BMP and other signals in yak hair follicles at different ages and between yak and other model animals is also a direction for further research in the future. We analyzed the differential gene expression of DP cells, and found that these genes were differentially expressed in different DP cell cycles, and reflected the differences in time and space, which provided an important reference for future research.

From the GO enrichment entries, it can be seen that DP cells are enriched in pathways such as the cell–cell junction, laminin binding, protein–DNA complex assembly, and protein–lipid complex, indicating that dermal papilla cells are responsible for the expression and transmission of genetic information. By analyzing the different branches, it was found that the first branch of dermal papilla cells mainly focused on cell proliferation and the function of participating in cell junction information transmission, which also reflected the importance of DP cells as the control center of hair follicle growth. DP cells can subtly control the continuous growth of mammalian hair by releasing a large number of molecular signals [88]. During the whole hair cycle, the DP is in a favorable position, where it can provide induction signals and guide the cell activity of hair follicles. The second branch focuses on the pathways of cell differentiation, hair cycle and angiogenesis. These pathways may be involved in the downward migration of hair follicles from telogen to anagen to the dermis, which is connected with capillaries and provides nutrients for hair growth. In the third branch, not only were cell connection information transmission and other related functions enriched, but apoptosis-related functions were also enriched in the second gene set. Yang et al. [32] found that the second branch of cashmere goat DP cells was enriched in the apoptosis process via a pseudotemporal analysis of cashmere goat DP cells. They speculated that the regulation of normal gene transcription into pseudogenes in the process of apoptosis coordinated hair follicle apoptosis. We analyzed the differential gene expression and potential functions of DP cells, but more details, such as the spatial distribution pattern of gene expression in DP cells, have not been studied and described in detail in mice and yaks. Future research may focus on such a theme, which may provide new insights for our subsequent research on hair follicle biology and hair follicle regeneration.

A CellChat analysis revealed a high number and intensity of interactions between RF and other cell types in the dermal fibroblast lineage. Notably, RF was identified as the most important cell for SPP1 signaling pathway output. This means that SPP1 plays an important role in fibroblasts and shows high expression characteristics under matrix activation, which further promotes the high expression of fibrosis genes [89]. By using Tyr-NrasQ61K; Spp1−/−mice, researchers found that SPP1 loss-of-function mutations are sufficient to reverse the static hair cycle in the skin of congenital nevus and that SPP1 can induce new hair growth [90]. Periodic hair growth and dormancy are complex processes regulated by a variety of internal and external factors, and SPP1 is a key mediator, which indicates that high expression of SPP1 in fibroblasts may play an important role in regulating hair cycle and wound repair. Liaw et al. [91] confirmed the role of SPP1 in the process of wound repair; by simultaneously knocking out the SPP1 gene in the mouse skin incision model, it was observed that the matrix disintegration and collagen fiber diameter decreased and the incision could not be repaired, suggesting the importance of OPN in the process of wound repair. In addition, inflammatory-cell-mediated signaling can trigger the upregulation of SPP1 in wound fibroblasts, which may delay the repair process and may also partially lead to fibrosis caused by wound healing [92].

## 5. Conclusions

In summary, this study used scRNA-seq technology to draw a cell transcription map in the skin, systematically described the changes in the expression of time-like genes during dermal fibroblast lineage and DP cell specialization, constructed an intercellular communication network, and demonstrated the interaction between identified cell types. The current research also highlights the previously neglected cell fate determination process in the cycle of yak hair follicles. In addition, by dissecting the heterogeneity of fibroblast subtypes, our study highlights that different fibroblast signals coordinate the asynchronous development of different cell types. The results of this study can be used to promote further research on the molecular regulatory mechanism of hair follicles and provide new insights into the functional analysis of dermal cell lineage during the transition of yak hair follicles from telogen to anagen.

## Figures and Tables

**Table 1:** Single-cell data set quality statistics.

Sample	Telogen	Anagen
Estimated number of cells	14,573	12,000
Median genes per cell	1261	927
Genome reads mapped	87.6%	93.3%
Valid barcodes	97.7%	97.1%
Total genes	17,796	18,420

## Data Availability

All the sequencing data used in this research are deposited in NCBI GEO databases under accession number: GSE205456. Data on telogen are scheduled to be released on 30 May 2024.
